# Symptomatic Pneumomediastinum Following Laparoscopic Cholecystectomy: A Case Report and a Literature Review

**DOI:** 10.7759/cureus.24604

**Published:** 2022-04-29

**Authors:** Hani H Maalouf, Ribal Aby Hadeer, Omar Tabbikha, Christina Abou-Malhab, Raja Wakim

**Affiliations:** 1 General Surgery, Mount Lebanon Hospital Balamand University Medical Center, Beirut, LBN

**Keywords:** spontaneous pneumomediastinum, isolated pneumomediastinum, case report, laparoscopic cholecystectomy, pneumomediastinum

## Abstract

Isolated pneumomediastinum is a rare complication after laparoscopic procedures. Herein, we present a case of a 38-year-old woman who presented two days after laparoscopic cholecystectomy with pleuritic chest pain and dyspnea and was found to have isolated pneumomediastinum. The patient was admitted for monitoring, oxygen therapy, and antibiotic prophylaxis and she was discharged on the fourth postoperative day when her symptoms resolved both subjectively and radiologically. Only two other cases of symptomatic isolated pneumomediastinum after laparoscopic cholecystectomy were reported in the literature and all of them were female patients, diagnosed radiologically, and treated conservatively. Therefore, isolated pneumomediastinum should be included in the differential diagnosis of dyspnea and chest pain after laparoscopic surgeries in order to have an early diagnosis, start early treatment, and prevent unnecessary investigations or advancement of the disease.

## Introduction

The treatment of choice for symptomatic cholelithiasis has become laparoscopic cholecystectomy [1]. Laparoscopic surgeries have gained wide acceptance in our time due to the reduced morbidity and mortality, shorter hospital stay, better postoperative recovery, less postoperative pain, and reduced cost as compared to open surgeries [2]. Despite its advantages, it may still have complications, namely common bile duct injury, bowel injury, liver injury, bleeding, and bile leak [3].

Pneumomediastinum is characterized by the unusual presence of air in the mediastinal cavity. It is an infrequent complication subsequently to laparoscopic abdominal surgeries in general with only two cases reported in the literature of an isolated pneumomediastinum after laparoscopic cholecystectomy [1,4]. We present the case of readmission of a patient with pleuritic chest pain following laparoscopic cholecystectomy who was found to have isolated pneumomediastinum. We also review the literature discussing presentation, diagnosis, etiology, and treatment options.

## Case presentation

A 38-year-old woman with a history of recurrent urinary tract infections presented initially with fever, abdominal pain, and vomiting, and was found to have pyelonephritis. The patient was admitted for medical management with broad-spectrum antibiotics. During her hospital stay, she started experiencing postprandial right upper quadrant pain of two days duration. Vital signs were stable. Laboratory tests were unremarkable with mildly disturbed liver function tests. Abdominal ultrasound showed an adequately distended gallbladder with multiple stones with the largest stone measuring 7 mm and no sign of acute cholecystitis. The patient was diagnosed with symptomatic cholelithiasis and the decision to proceed with laparoscopic cholecystectomy was taken.

**Figure 1:**
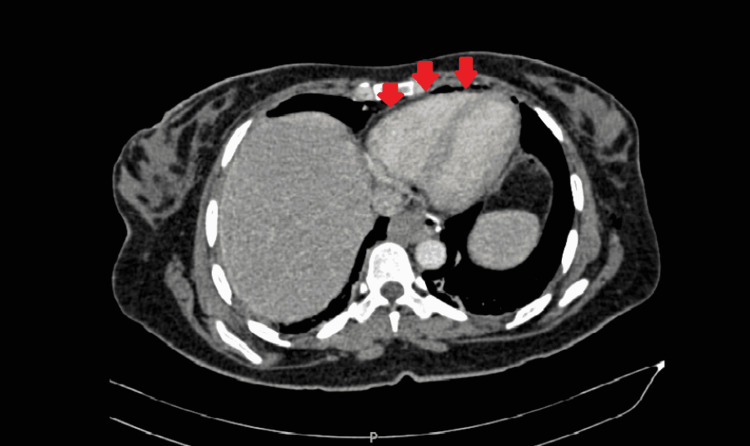
Chest CT Scan Showing Pneumomediastinum Red arrows pointing to rim of air are seen within the mediastinum. CT, computed tomography.

**Figure 2:**
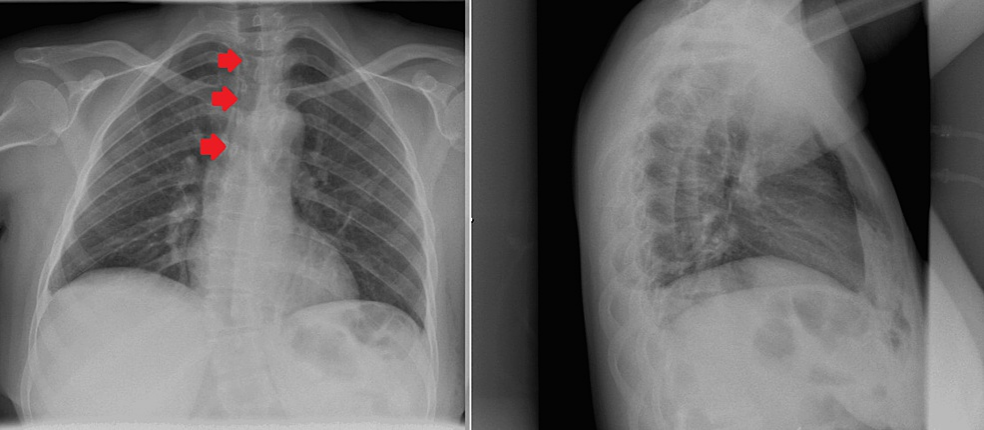
Chest X-ray Showing Pneumomediastinum Red arrows point to air in the mediastinum.

Surgery was initiated with insufflation of the peritoneal cavity with carbon dioxide via Veress needle and insertion of trocars. Veress needle was inserted at Palmer’s point with a single successful attempt and no complications. As for the trocars, four trocars were inserted smoothly with no complications at the umbilical, epigastric, left subcostal, and right subcostal regions. An optimal pneumoperitoneum pressure was set at 13 mmHg and the duration of the operation was 42 minutes. A critical view of safety was adopted with dissection of the hepatobiliary triangle and clipping of the cystic artery and duct. The surgery was smooth and no intraoperative complications were observed. The patient was discharged on the first postoperative day. On the second postoperative day, the patient presented to the emergency department complaining of right upper quadrant pain, dyspnea, and chest pain on deep inspiration. The patient was afebrile and her vitals were stable including oxygen saturation. Laboratory tests were unremarkable and arterial blood gases were normal. Chest abdomen pelvis CT (computed tomography) scan showed clear lungs, pneumomediastinum dissecting into the soft tissues of the neck, where it is exerting mass effect on the trachea. No signs of subcutaneous emphysema, pneumothorax, tension pneumomediastinum, mediastinal shift, pneumoperitoneum, pneumoretroperitoneum, or other intra-abdominal collections were noted (Figure 1). Chest X-ray showed the same findings (Figure 2). There was no suspicion of pulmonary embolism considering the clinical hemodynamic picture of the patient, so no further investigations were undertaken. 

Our patient was admitted for pain management and was started on oxygen therapy since she was dyspneic. Antibiotics were initiated to prevent mediastinitis. On the third postoperative day, the patient improved clinically and the chest X-ray showed a decrease in pneumomediastinum. On the fourth postoperative day, the patient was no more complaining of chest discomfort and was off oxygen, chest X-ray improved significantly, and she was discharged home. The patient was seen two months after discharge and she was still asymptomatic; therefore, no further investigations were requested.

## Discussion

The definition of pneumomediastinum is the presence of air inside the mediastinal cavity [1]. Hamman was the first to describe spontaneous pneumomediastinum in 1939, and his name was attributed to the hallmark sign of this condition [1,4]. Spontaneous pneumomediastinum is not considered to have a causative factor as compared to secondary pneumomediastinum, which may be caused by trauma, such as blunt or penetrating injuries to the thorax or the abdomen; lung diseases, such as asthma, bronchiectasis, chronic obstructive pulmonary disease, and inhalation of toxic gases; or iatrogenic procedures including intubation, bronchoscopy, and endoscopic procedures [4,5].

Carbon dioxide insufflation during laparoscopic surgeries although safe and blood soluble is associated with subcutaneous emphysema with a reported incidence of 0.43% to 2.34%, pneumothorax of 0.03%, and pneumomediastinum of 0.02%. The actual incidence of these complications may actually be much higher due to the inability to the diagnosis of these conditions that may be asymptomatic in many of the cases [2].

It is speculated that some risk factors for the development of pneumothorax or pneumomediastinum during laparoscopic surgery are an extensive operative time of 3 hours or more, elder patients, the higher maximum measured end-tidal CO_2_ (>50 mm Hg), hiatal operations such as fundoplasty, operator inexperience, and the use of six or more surgical ports [6]. In the case of our patient, none of these risk factors were present.

Very few cases are reported to date of postoperative pneumomediastinum and even fewer of symptomatic isolated pneumomediastinum after laparoscopic cholecystectomy, namely two previous cases, were only reported in the literature found after extensive research in PubMed [1,4] (Table 1). Female predominance is noted. All had their symptoms appear shortly postoperatively. The presentation of pneumomediastinum may be broad and sometimes vague. It may range from chest pain, shortness of breath, desaturation, abdominal pain, hypotension, and fever to being asymptomatic, so a high clinical suspicion should be kept in mind in order not to miss it.

**Table 1:** Cases of Isolated Pneumomediastinum Following Laparoscopic Cholecystectomy LC: Laparoscopic Cholecystectomy; RUQ: Right Upper Quadrant; CT: Computed Tomography; N/A: Not Applicable; CRP: C-Reactive Protein; postop: Post operation.

Reference/Year	Age/Gender	Type of Surgery	Symptoms	Postoperative Day of Presentation	Diagnostic Test	Laboratory Findings	Treatment	Day of Discharge
Kostis et al. [1]/2019	47/F	LC	Dyspnea, nausea	1	Chest CT	N/A	Oxygen therapy, antibiotics	5 postop
Aydin et al. [4]/ 2014	51/F	LC	Respiratory difficulties, temperature 38°C	1	Chest CT	Elevated white count and CRP	Intensive respiratory physiotherapy, antibiotics	14 postop
Present case/2021	38/F	LC	RUQ pain, pleuritic chest pain, dyspnea	2	Chest CT, chest X-ray	Normal	Oxygen therapy, antibiotics	4 postop

Chest X-ray and chest CT are the modalities of choice for diagnosis. A radiolucent air-line is usually noted in the mediastinum and surrounding the heart [4]. In the case of Aydin et al., chest X-ray failed to show the pneumomediastinum, and chest CT was the diagnostic test, whereas in our case both chest X-ray and CT showed the pathology, so a combination of both is advised to be done. Not to mention that chest CT can also rule out other acute complications that may develop postoperatively including pulmonary embolism or pneumonia. Laboratory findings range from normal to increased inflammatory markers. In the latter situation, clinical suspicion of mediastinitis should be kept in mind, and ruling out other infectious conditions should be done.

Diagnosis should be made quickly followed by appropriate treatment. The patient should be hospitalized for at least 24 to 48 hours for better monitoring. Supplementary oxygen showed to be effective for pneumomediastinum and was used in all cases. It helped with dyspnea and desaturation and allowed better oxygenation. Chest physiotherapy can be used in more severe cases or in cases not responsive to oxygenation. Antibiotic treatment may be used in cases of fever or infection, or to prevent mediastinitis [1,4]. Serial chest X-rays may be needed for better follow-up or a repeat chest CT can be done before discharge in order to assure resolution of pneumomediastinum.

Generally, the etiology of pneumomediastinum is not easily determined. One theory is the rupture of emphysematous bullae due to barotrauma during mechanical ventilation. The bullae are more typically located at the hilum of the lung. This may be spontaneous or due to tobacco or recreational drug use. It can also be due to direct iatrogenic injury to the diaphragm during a surgery, or defects of the diaphragm including the anatomical hiatus, or the presence of diaphragmatic congenital channels, or diaphragmatic congenital weak points [1,4,7]. Delgado-Plasencia et al. reported a case of pneumomediastinum due to emphysematous cholecystitis. Emphysematous cholecystitis is a form of acute cholecystitis that is usually caused by gas-forming organisms. The gas will spread within the subcutaneous tissue and peritoneal cavity and can reach the mediastinal cavity [8].

In our case, it was hard to decide the exact way of the migration of the carbon dioxide to the mediastinum. Neither damage or injury to the diaphragm, nor barotrauma during ventilation were reported as intraoperative complications. Our case is similar to the two cases reported previously that reported no intraoperative complications, and we hypothesize similarly that isolated pneumomediastinum without any evidence of subcutaneous emphysema was the result of the passage of carbon dioxide retroperitoneally to the mediastinum from diaphragmatic or esophago-aortic hiatuses [1,4]. 

No major clinical consequences are feared from pneumomediastinum alone. Richard et al. studied the impact of extra-alveolar air collections after laparoscopy and found that pneumomediastinum with or without pneumothorax was not related to significant morbidity [9]. However, a tremendously large amount of mediastinal air can lead to cardiorespiratory distress. This happens in very rare cases and it is primarily due to the normal return of venous blood to the heart [10]. Tension pneumomediastinum is a life-threatening situation that may lead to a decreased cardiac output. This is either due to direct cardiac compression from a full mediastinum or due to reduced venous return, in addition to airway compression. Tension pneumomediastinum should be quickly handled by video-assisted thoracoscopic surgery or even by thoracotomy. In some cases, pneumomediastinum may further be complicated by a concomitant pneumothorax or extensive subcutaneous emphysema or even expand to the retropharyngeal space that should always be handled by the right measures [11].

## Conclusions

Isolated pneumomediastinum is a rare finding after laparoscopic cholecystectomy. A high clinical suspicion should be confirmed with radiological imaging since sometimes it is asymptomatic and in others may present with vague symptoms. In-hospital monitoring should be assured with appropriate oxygenation and antibiotic coverage to prevent serious life-threatening outcomes such as mediastinitis and tension pneumomediastinum. Anesthesiologists, surgeons, and re-animators ought to be aware of these complications all through laparoscopic procedures in order to have immediate and suitable treatment.
